# “My everyday life has returned to normal”- Experiences of patients and relatives with a palliative day care clinic: a qualitative evaluation study

**DOI:** 10.1186/s12904-023-01140-5

**Published:** 2023-03-17

**Authors:** Anne Müller, Alfred Paul, Johannes Best, Stephanie Kunkel, Raymond Voltz, Julia Strupp

**Affiliations:** 1 Department of Palliative Medicine, Faculty of Medicine and University Hospital, University of Cologne, Cologne, Germany; 2 Clinical Center Aschaffenburg-Alzenau, Alzenau, Germany; 3 Center for Integrated Oncology Aachen Bonn Cologne Duesseldorf (CIO ABCD), Faculty of Medicine and University Hospital, University of Cologne, Cologne, Germany; 4 Center for Health Services Research, Faculty of Medicine and University Hospital, University of Cologne, Cologne, Germany

**Keywords:** Palliative medicine, Supportive care, Patient care, Relatives, Day care, Medical, Qualitative research

## Abstract

**Purpose:**

Palliative day care clinics (PDCCs) complement inpatient and home palliative care and provide access to a range of multi-professional services. However, they are not part of standard care in Germany. Yet, international studies show that PDCCs have a positive impact on e.g. quality of life.

To evaluate one of the first PDCCs in Germany (Aschaffenburg-Alzenau (PDCC-AA)) by describing the experiences, satisfaction, challenges, wishes of patients and relatives and possible alternatives to treatment in the PDCC.

**Methods:**

Qualitative study using semi-structured telephone interviews. Data was analyzed using qualitative structuring content analysis according to Kuckartz with deductive a priori categories and inductive subcategories.

**Results:**

A total of 31 patients and 38 relatives completed telephone interviews. The majority of patients were diagnosed with a cancer or tumor disease. The following four main themes emerged: (1) alternatives to treatment at the PDCC, (2) symptom relief, (3) sense of security, (4) “everyday life framing” (normality of everyday life).

Participants valued the medical treatment (especially for pain), psychosocial support given and having direct access to a range of services (e.g., wound care and pleural drainage), while relatives valued being provided respite services. A sense of security, availability of therapies, and devoted time that healthcare providers spent to explain e.g., treatment options were mentioned most positively, as well as confidence in dealing with the illness. As to whether there was an alternative to treatment in the PDCC, some saw further inpatient stays, the emergency room or care by general practitioners as options (although not preferred). Patients expressed concern that they were not treated and informed according to their needs in other care settings.

**Conclusions:**

PDCCs may close a gap between inpatient and home palliative care. Participants mentioned that hospital stays can be delayed or even prevented.

## Introduction

Palliative day care clinics (PDCCs) are well established in the UK, with the first service units opening in 1975 [1], but a standardization of PDCCs is still lacking [2, 3]. In Germany, also, they are not part of standard care. A current analysis by Apolinarski et al. (2021) shows that eight PDCCs could be identified, of which three clinics are currently still under construction [4]. Similarly, there is a paucity of studies on PDCCs in English- and German-speaking countries.

The aim of a PDCC is to achieve improved symptom control in patients, who are seriously ill and have palliative care needs, through medical (invasive) therapies and psychosocial treatment, resulting in improved quality of life by providing multi-professional holistic care. If possible, inpatient hospitalization should be delayed or prevented and generalist palliative home care (GPHC) and/or specialist palliative home care (SPHC) should be supported [5]. In day hospices the focus is structuring the day of patients [4].

PDCCs are a specialized service for patients who are seriously ill, living at home and do not require 24-hour hospital care. In the (day) hospice, patients receive care from their primary care physician as needed. However, the rationale of a PDCC is to provide specialist care and (invasive) treatments such as blood transfusions, ascites punctures, and wound care. The intention is to provide symptom control for the seriously ill patients living at home. Patients of a PDCC are assigned by treating physicians (e.g., in the hospital or family physician), by a palliative home care service (GPHC/SPHC) or a treating specialist physician. The current PDCCs in Germany can be visited between 1 and 5 days a week at a core time between 10 am and 3 pm according to the needs of the patients. PDCCs are usually affiliated with a hospital in order to be able to use its diagnostic and therapeutic possibilities, as well as a multi-professional team [4]. Both the spatial/interior design and human resources are geared to the special needs and wishes of seriously ill and dying people. Comprehensive and holistic care is offered with the help of full-time and volunteer staff [5]. The main advantage of a PDCC is the ability to offer multidisciplinary services under one roof where patients are “visited” by healthcare services. In contrast, a day hospice does not require a connection to a hospital, as it does not offer diagnostics and invasive medical treatments.

Especially in Germany, there is a lack of studies on the effectiveness and differentiation from other forms of palliative care provision (e.g., PDCCs). The German S3 guideline for palliative care aims to ensure optimal palliative care for patients diagnosed with an incurable disease. The leadership of the German Society for Palliative Medicine (DGP) developed the guideline within the methodological framework of the German Guideline Program in Oncology [6, 7]. Research results analyzed in the S3 guideline for palliative care on PDCCs abroad, show positive effects on patients receiving palliative care [5].

A Belgian study on PDCCs found that they offered unique services for patients, but adequate funding was lacking and referral rates needed to increase to “unlock” the potential of palliative day care services [3, 8]. Research on the potential benefits of PDCCs is missing, which may cause this healthcare service to be underappreciated. Especially regarding the early integration of day services alongside other models of care, PDCCs have the potential to provide early access to palliative care [2]. But, due to the lack of standardization and poor visibility of the service among healthcare professionals, more research is needed.

Therefore, the aim of this qualitative sub-study is to evaluate one of Germany’s first PDCCs by qualitatively exploring the satisfaction of patients and relatives, challenges, treatment trajectories, ways of getting attentive to the PDCC and possible alternatives to a PDCC.

## Methods

This study is part of a larger evaluation study. Here, within this publication we present the qualitative data collected within semi-structured telephone interviews with patients and family members. Data was collected between November 2020 and April 2021. This study is reported following the Consolidated Criteria for Reporting Qualitative Research (COREQ) guidelines. Analysis of routine data as a quantitative part of the evaluation study will be published elsewhere.

### Setting

The PDCC-AA was founded in 2012 to complement the eight bed inpatient palliative care unit at the Aschaffenburg-Alzenau Clinic. It consists of four single patient rooms and the facilities of the palliative care unit. The multidisciplinary team members represented are doctors, nurses, social workers, physiotherapists, psycho-oncologists, pastoral care workers and volunteers from a hospice group. The infrastructure, diagnostic and treatment facilities of the entire Clinic can be used as needed.

Patients visit the PDCC in different frequencies, from 1 up to 5 days a week. Some patients visit the PDCC-AA on a monthly basis or less only. All patients have an appointment with the attending physician at PDCC each time they visit PDCC. All further therapies and potentially (invasive) measures as well as further treatments and diagnostics in the nearby hospital are scheduled on an individual basis.

### Participants

Individuals were considered eligible if they were currently being treated at the PDCC-AA or had been treated there.

Eligible participants (patients and their relatives) needed to be aged over 18 years and able to give informed consent. Following the ethics relatives could only participate if the patient had agreed. Relatives were defined as persons involved in the patient’s care and/or of special importance for the patient.

**Fig. 1:**
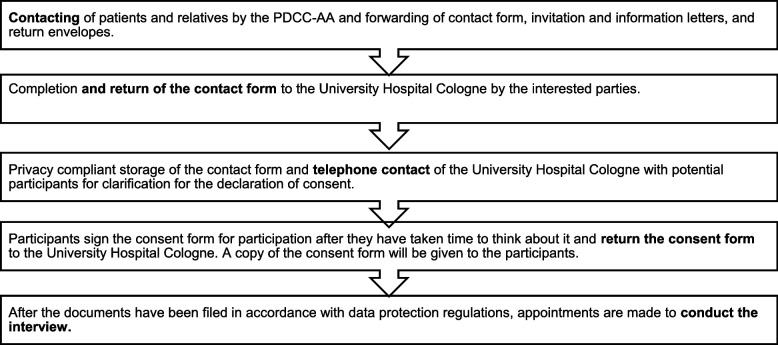
Recruitment process

Due to the Covid-19 pandemic eligible participants were contacted by the first author (AM) via telephone after they had been informed about the study by a physician of the PDCC-AA and provided consent that their contact details could be given to the researchers. This may have biased the results (see Fig. 1 for the recruitment process).

We received a total of 80 contact requests from patients and relatives. Seventy-five send us their signed consent forms. A total of 69 interviews were conducted. Reasons for non-participation were: In four cases, relatives did not want to participate, and one relative could no longer be contacted. Two patients died, two could no longer be contacted, and two did not participate in the interview without giving a reason. Thirty-one patients (mean age 66.5) and thirty-eight relatives (mean age 60) completed telephone interviews between November 2020 and April 2021. Interviews lasted between 9 and 69.5 min (mAvg = 28 min).

### Data Collection

Semi-structured interviews were carried out via telephone and recorded. Go To meeting Software was used to record the data according to ethical guidelines and data safety. Mealer et al. (2014) describe that a relationship and connection with participants can also be established by telephone. In addition, privacy is protected. Furthermore, the effort for participants is low [9].

A topic guide was developed by the research team (consisting of clinical experts, health services researchers) in charge of the two authors (AM and JS – Research Coordinator, PhD in health sciences). It includes relevant aspects for the evaluation, complemented by a preceding short quantitative survey (questions on sociodemographics - covering age, gender, nationality and living situation, additional care provided from GP, hospice services, meals-on-wheels, SPHC, GPHC, nursing care levels and if an inpatient hospital stay could be delayed or avoided by staying in the PDCC). To enhance credibility, the topic guide was pre-tested and meticulously discussed with research and clinical staff within a research workshop.

In the end, it contained the following guides, same for patients and relatives:

**Table:** 

• What symptoms were crucial and burdensome for you to seek treatment at the PDCC?
• What would have been the alternative to treatment at the PDCC if the PDCC had not existed?
• How have your symptoms changed as a result of your treatment at the PDCC?
• How do you experience your treatment at the PDCC?
• In what way do you think the treatment at the PDCC is helpful for you?
• How has your treatment at the PDCC affected your relatives?
• What expectations did you have of your treatment at the PDCC and to what extent were they met?

Probing questions were used to get more specific and in-depth information. Interviews were completed by one author (AM, female, research associate, M.A. degree in rehabilitation sciences) who had no prior relationships with patients or relatives.

### Data analysis

**Fig. 2:**
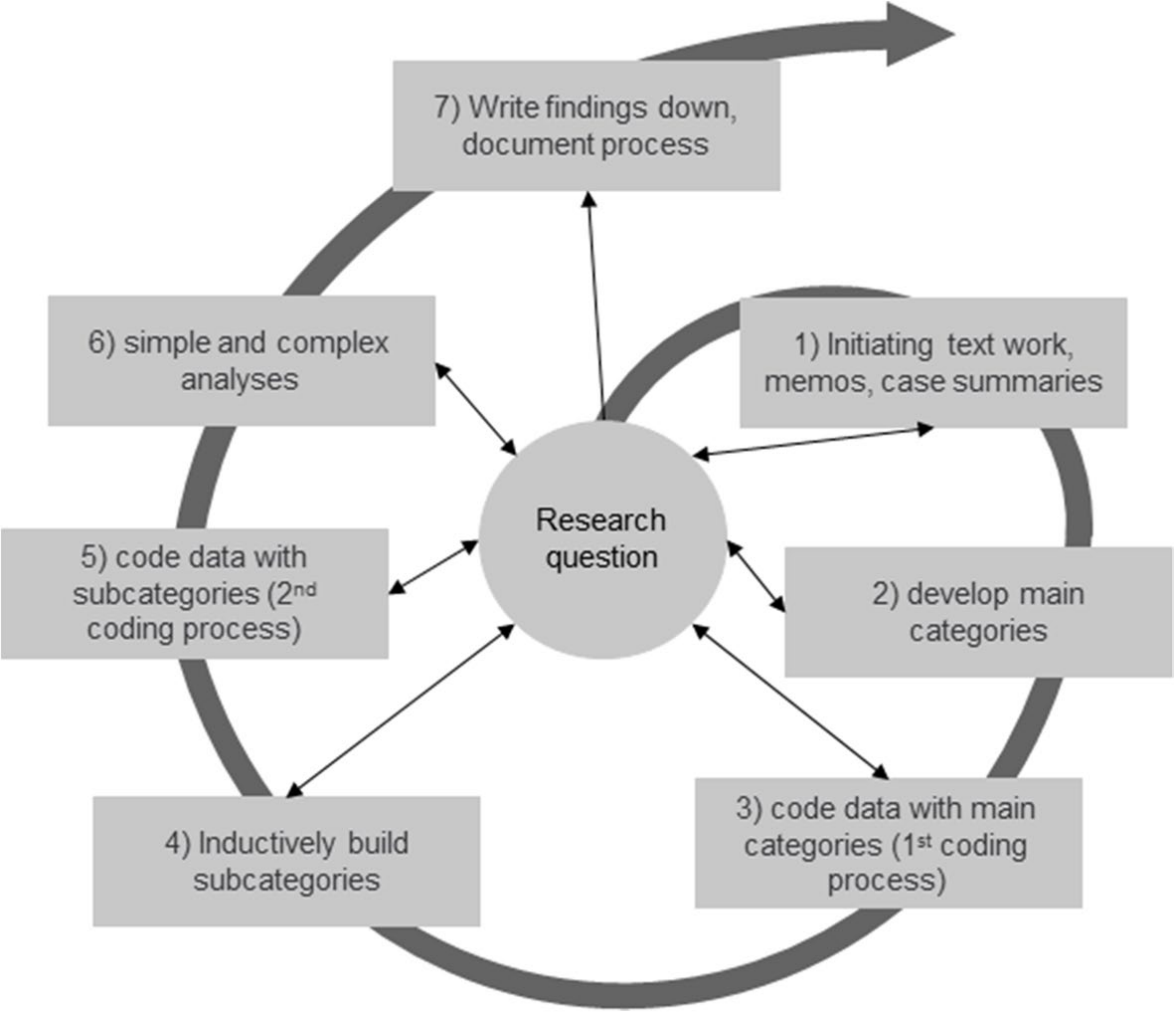
Qualitative structuring content analysis (Kuckartz 2022) [10] translated by AM

We analyzed the preceding questionnaire using program SPSS and descriptive statistics. Audio recordings were transcribed verbatim externally and managed using MAXQDA 2020. In the course of the transcription, data was anonymized. This included sensitive information, such as personal names, dates of birth, diagnoses or addresses. The transcript was titled with a pseudonym composed of an arbitrary combination of numbers and letters that did not allow any conclusions to be drawn about a person. The audio files were safely stored in a data protection folder. Transcripts were not returned to the participants. We analyzed the data following the qualitative structuring content analysis method according to Kuckartz (2018) [10]. As illustrated in Fig. 2, the qualitative structuring content analysis was conducted. Initially, the first author (AM) read and reread the transcripts to gain a sense of the experiences of each patient and relative. The first Author (AM) developed a priori and deductively a category system based on existing literature. Whenever relevant text segments could not be classified according to the existing categories and corresponding subcategories, new subcategories were created [10, 11]. JS familiarized herself with a random sample of the responses. Afterwards, the codes and categories were discussed and negotiated between the authors (AM and JS) to reach a consensus and adjustments were made if necessary. A peer debriefing was additionally conducted to probe the process and establish credibility and enhance validity. Transcripts were not returned to participants for comment or correction. We derived categories deductively following the process of qualitative structuring content analysis. They are oriented on the objectives of the evaluation, which are to find statements on satisfaction of patients and relatives, challenges, treatment trajectories, ways of getting attentive to the PDCC and possible alternatives to a PDCC.

### Ethical considerations

Participants were provided with oral and written information about the study and provided written informed consent. Ethical approvals were obtained from the Ethics Committee of the University of Cologne [#20-1377].

## Results

### Preceding short quantitative survey

Of the 31 patients, 29 had statutory health insurance and two of them had private health insurance. Table 1. shows the living situation of patients during treatment at the PDCC-AA.

**Table 1.** Living situation during treatment at the PDCC (multiple answers possible)

Living situation	n
With partner	20
Alone	9
With other person	5
In care facility	1
Other living situation	2
With child	1

The main diagnosis was cancer (83.9%), followed by cardiovascular disease (9.7%) and other (6.5%). About 64.5% (20 out of 31) of patients stated that inpatient hospitalization could have been avoided.

### Qualitative data

We have extracted the four following categories deductively : (1) alternatives to treatment at the PDCC, (2) symptom relief, (3) sense of security, (4) “everyday life framing” (normality of everyday life). The citations are cited with age of the interviewee and diagnosis of the referring patient and numbers and letters. A number indicates that the citation is from a patient (e.g., 1002); a number and a letter indicate that the citation is from a relative (e.g., 1003B).

### Alternatives to treatment at the PDCC

As one objective of the evaluation, this category was deductively derived a priori. It was divided into the following three inductively built subcategories:

- referral,
- expectations and fears,
- alternatives to treatment at the PDCC.

#### Referral

Patients and relatives described how their path to the PDCC began. A total of 32 statements in the interviews with relatives and eight in the interviews with patients were assigned to this subcategory.

Patients reported that effects of chemotherapy and related recommendations of the responsible oncologist or referral after an inpatient hospital stay were their ways to the PDCC.

Relatives reported other “access routes” to the PDCC. Some reported that contact with the PDCC came about through “snowballing” (e.g., contacts who had experience with the PDCC or had heard about it before).

“It came from my sister, who in turn was being treated by a physiotherapist for her cancer, (…). And then this lady said, so whether that wouldn’t be, yes, to consider, that he introduces himself to the PDCC”. (1028B, 52y, diag, of pat.: cancer, para. 2)

Also, family physicians were frequently mentioned.

“And then she discussed it with her family doctor. And this family doctor then recommended this PDCC to her”. (1037B, 54y, diag. of pat.: cancer, para. 5)

Furthermore, according to the statements of relatives, many patients were made aware of the possibility of the PDCC on various wards of the Aschaffenburg-Alzenau Clinic.

“So then we were made aware of the PDCC by the clinic, also for the reason that my father had strictly refused to be treated as an inpatient. He wanted to avoid by ‘hook or by crook’ being treated as an inpatient in a hospital”. (1047B, 41y, diag. of pat.: cancer, para. 5)

#### Expectations/Fears

All statements dealing with expectations and/or fears were assigned to this subcategory. In total, patients made 32 statements and relatives made 21 statements on this topic. Patients often stated that they had no expectations of the PDCC at first.

“Well, if I’m completely honest, I didn’t really have any expectations at first, because I first thought, hm, let’s see what happens there anyway (…)”. (1032, 61y, diag.: cancer, para. 55)

Most reported having hope for symptom relief or improvement in quality of life before visiting the PDCC.

“Expectations, yes, that it would get better with the water in the lungs. That was my hope”. (1009, 64y, diag.: cancer, para.105)

Many stated that they had hoped for support and contact persons.

“Insofar as expectations, (…) that is the expectation that I have a competent contact person on topics where one simply has no contact person in the other hospital business or health business due to lack of time”. (1005,63y, diag.: cancer, para. 54)

Many were also sceptical about the PDCC or imagined something different under the term “palliative” than they then experienced at the PDCC.

“And that’s when I got scared at first, said ‘Oh dear, the last stop (…)’. They said ‘No, that has nothing to do with it at all’ and told me about their services, and I was also pleasantly surprised after the first treatment”. (1008, 73y, diag.: cancer, para. 96)

It is also clear from the reports of the relatives that there was often a startled and frightened reaction to the word “palliative”.

“‘There is also the possibility to go to the palliative care clinic’, which was like a shock at first, yes, because it was- ‘palliative’ is such a thing, that one avoids (…)”. (1030B, 61y, diag. of pat.: cancer, para. 7)

#### Alternatives to treatment at the PDCC

As a third subcategory, alternatives to treatment at the PDCC were enumerated and assigned in further subcategories. Patients often saw no alternative.

“There weren’t so many options, especially from the, yes, from the type and process of how the treatment in the day clinic is. I hardly experienced that otherwise, or nowhere”. (1007, 72y, diag.: cancer, para. 15)

One patient feared being alone without support at home.

“There I would have remained alone with my fear and would also be here in this house and would probably have more medication consumption (…)”. (1054, 74y, diag.: cancer, para. 7)

Receiving care by established specialists was mentioned as a further alternative.

“I believe, then my dad would have nothing else, just the doctors, the “normal” doctors, where one comes and goes, to the urologist and to the lung doctor. And yes, then he would have just run on from doctor to doctor, I say”. (1052B, 34y, diag. of pat.: cancer, para. 13)

The patient’s own GP was also named as an alternative.

“(…) or it should have been done by the family doctor, but I don’t know whether he knows so much about metastases, about bone pain, because in the PDCC there are real experts (…)”. (1018, 58y, diag.: cancer, para. 5)

Also, the emergency room of the hospital would have been the alternative to the PDCC for some.

“And I then, I think, two or three times he even referred me to the emergency room, where I felt very out of place, because I think an emergency room is a completely different thing when people are taken from the highway by helicopter or something else than like a woman sitting there who has a big belly and can hardly move. So I always felt very out of place there, (…), the first time I was there, I waited nine hours to be seen by the doctor”. (1017, 69y, diag.: liver cirrhosis, para. 4)

### Symptom Relief

All statements dealing with symptoms and their changes over the treatment period were assigned here. The a priori built category of symptom relief was further subdivided into three inductively built subcategories:

- decisive symptoms,
- perceived helpfulness of treatment,
- changes in symptoms and problems.

#### Decisive symptoms

In this subcategory, all statements about symptoms prior to admission to the PDCC were recorded. Patients themselves and relatives predominantly stated that severe pain was the decisive factor.

“Insane pain all over the body. Nerve pain at night, my legs, everything hurt me, really everything. And it actually forced me to, let’s say, do something. And that’s when I went to the day clinic, yes, for that reason”. (1033, 68y, diag.: fibromyalgia, para. 5)

One relative also saw the pain symptomatology from the patient as decisive, which is in connection with the early integration in what she sees as a suitable form of care.

“Mainly pain management, and also knowing that the treatment options are reducing more and more. He can’t do chemo indefinitely anymore, and there will be little therapy still available afterwards”. (1055B, 45y, diag. of pat.: cancer, para. 5)

Psychological problems were also named as a decisive reasons to come to the PDCC.

“So it was like, yes, we just couldn’t cope at home anymore, I must say. So the pain was getting worse and worse. The psyche became more and more unstable”. (1033B, 41y, diag. of pat.: fibromyalgia, para. 8)

It was further stated that the need for invasive measures was decisive.

“Well, as I just told you, it was just that, this ascites, this accumulation of water in the abdomen, which becomes more with each day and I can then also walk worse, get less air, it was just decisive to present me in the palliative day clinic to have the ascites sucked out, yes”. (1017, 69y, diag.: liver cirrhosis, para. 2)

#### Perceived helpfulness of treatment

There were frequent statements about whether treatment in the PDCC was considered helpful. It was reported that stays in the PDCC allowed the patients to deal with their illness in a protected space.

“(…) when he comes home, (sighs), he is a bit more (…) calmer, because it is again a different - a different way of dealing with the topic of illness, with the topic of death, with the - than it is handled in the family and he can also simply come to rest there probably (…) and at the same time is cared for”. (1055B, 45y, diag. of pat.: cancer, para. 9)

Psychosocial care and therapy were also frequently described as supportive.

“Well, because, yes, I say so, I just feel comfortable. I don’t go there on Friday with any uneasiness, but I go there with a joyful feeling, because I know I’m in good hands there and I can discuss everything with anyone at any time (…). That alone is a good feeling for me”. (1003, 64y, diag.: cancer, para. 53)

Invasive measures were also seen as relieving and helpful.

“They pulled water out of me (…). I was a bit afraid of that. And I almost didn’t notice it at all. It worked for me too - I was quite surprised. And the water they let run out was three and a half litres the first time”. (1013, 69y, diag.: cancer, para. 68)

“The doctor can see if she has water in her lungs again, always does an ultrasound right away and that it doesn’t get as bad as it was last time. It’s just good for her, every 3 weeks to visit the day clinic”. (1004B, 69y, diag. of pat.: cardiovascular disease, para. 25)

Some statements were also made regarding pain therapy and medication adjustment.

“And there they have then also begun (…) this cannabis-therapy- and then additionally cortisone prescribed. And I have to say, that just stabilized his general condition a little bit. That did him good. And I don’t know if he hadn’t been in palliative care, whether another doctor would have done it that way”. (1022B, 67y, diag. of pat.: cancer, para. 21)

#### Changes in symptoms and problems

In this subcategory, all statements were taken into account that referred to the change in all types of symptoms and problems after admission to the PDCC. Patients made 39 statements and relatives made 64 statements. Improvement in (pain) symptomatology was mentioned the most.

“(…) Last year, in spring, she had so much pain, she was actually occupied with her pain the whole time, and assisted dying became a topic, you know. So, how long do you have to go through something like that? (…) And that has completely disappeared again as a topic, simply because she HAS less pain”. (1046B, 52y, diag. of pat.: cancer, para. 35–39)

In addition to medication, additional co-therapies were also described as helpful.

“They were super great therapists all around. So my wife has had a lot of problems with her back and she got really great massages right at the beginning, which really made it easier for her to move”. (1030B, 61y, diag. of pat.: cancer, para. 7)

Improvements in psychosocial issues were also reported very often.

“Yes, well, the complaints don’t go away until the liver somehow resumes its ‘duty’ or at least partially resumes. But it, yes, from the mental condition is just much much better, because I know, if something comes up, I can go there”. (1017, 69y, diag.: liver cirrhosis, para. 40)

### Sense of security

This category was derived deductively from the results of the evaluation by Schneider et al. (2015) [12]. All statements dealing with the aspect of security were included. Furthermore, we derived the following three subcategories inductively:

- time,
- comprehensive competence and responsibility,
- confidence in dealing with one's own illness.

#### Time

The aspect of time was also found in the analysis of Schneider et al. (2015) [12]. Patients particularly emphasized the time that medical staff took for detailed discussions.

“(…) and was then just pleasantly surprised that the doctors have time there, right, that it is not just a 5-minute consultation then, of ‘How are you?’. Aha, well, we’ll write this and that down, and then you’ll be fine and goodbye, ‘No, as I said, they have time or take their time (…)’”. (1044, 57y, diag.: cancer, para. 9)

The “time-factor” was especially seen as a source of security.

“(…) because I know there’s someone looking over all the things every month. I always take all my medical files with me and the blood work. And someone takes time and just looks over it, that everything fits, exactly, yes”. (1024, 48y, diag.: cancer, para. 21)

#### Comprehensive competence and responsibility

This subcategory was further subdivided into therapeutic services, social services support, and medical consultation. In total, 134 statements were made by relatives and 164 statements were made by patients in this subcategory. Regarding therapeutic services, it was frequently reported that creative therapies were rather helpful.

“The physio is a special story for me anyway, because I have metastases in almost all bones. So that helps me. The music therapy or art therapy, that are also- so there I was really very sceptical, because I’m not a musical person, (…) but I must say now, I would miss it very much, (…) that is totally relaxing, so I can totally switch off (…)”. (1055, 47y, diag.: cancer, para. 48)

Similarly, psychological support was described as supportive.

“The- yes of course, the psycho-oncology, there I can talk about all problems (…). I mean, you have problems already, first of all to process that you are suddenly incurably ill, the whole family situation, and it also affects the whole family at once. (…)”. (1018, 58y, diag.: cancer, para. 15).

Social services support was also mentioned.

“These whole, these whole things, where you normally have to fight, have to run, you know certainly, where you have to go everywhere, make applications, and then they check, and there will be this and there will be that. It’s much easier there. As I said, I would only have to say that I would like to have information about this and that, and then someone would come and advise me. So I think that’s also very very good, especially for someone who is seriously ill and now can’t go everywhere to Pontius and Pilate and introduce himself and-so it’s good”. (1003, 64y, diag.: cancer, para. 39)

The medical consultations and visits as a security-giving aspect in the sense of comprehensive responsibility were frequently mentioned by both patients and relatives.

“(…) that reassures me very much, because I know that this is my point of contact, which then provides me with relief and also ensures me a better quality of life at that moment”. (1020, 37y, diag.: cancer, para. 15)

“And that’s a whole other level of security, and my wife has that too, because when you know you have a contact person, they know you personally (…). And that is a completely different security, a completely different feeling than if you had to go to the hospital every time (…). And there it is just, there you know, either you have (immediately) a doctor at hand or they call back”. (1006, 55y, diag.: cancer, para. 49)

#### Confidence in dealing with one’s own illness

This subcategory refers to all statements on the topic of confidence in dealing with the disease. A total of 46 statements from patients and 76 statements from relatives were assigned to this category. Patients emphasized that the PDCC offered them a lot of confidence in dealing with their disease.

“(…) And I have become more confident now, so I also notice, (laughs) yes, exactly. (…) Yes, I hear- so the oncologist has just always prescribed and prescribed. And I just took it. And now I can rather, yes, give contra. (laughs) Yes, or ask whether one can reduce some medication, which I perhaps don’t necessarily need, yes, exactly”. (1024, 48y, diag.: cancer, para. 25–27)

Having a contact person in the PDCC in emergency situations also reassures many interviewees.

“(…) I was standing here in my kitchen and I had to sneeze and, yes, my stomach burst. (…) And the first thing I grabbed was my phone and called the palliative care department, and actually, yes, I could have been admitted to the hospital via the emergency room. But I was told to just come by”. (1017, 69y, diag.: liver cirrhosis, para. 26–28)

It was also reported that knowing that they were receiving regular treatment and symptom control increased their confidence in dealing with their own illness.

“It gives confidence just in our daily routine and my pain. The water in my stomach always comes back. And I just know that it will be gone in a week. It is predictable. That just gives me confidence again”. (1020, 37y, diag.: cancer, para. 41)

### “Everyday life framing” (normality of everyday life)

A priori this category was adopted from Schneider et al. (2015) [12]. Analysis resulted in the following two subcategories built inductively:

- perceived changes in everyday life
- effects on relatives.

In total, patients reported this 68 times and relatives reported this 145 times.

#### Perceived changes in everyday life

Patients and relatives often reported that treatment at the PDCC brought changes in everyday life, which were mainly based on the improvement of pain or other symptoms.

“(…) And then he was really relaxed. And that gave him strength for the whole week”. (1040B, 69y, diag.: cancer, para 0.24)

For some, the improvement in disease-specific symptoms led to positive changes in everyday life.

“My daily life has returned to normal. I was lying down a lot before. And because of that, I also had bedsores. And then this stupid fistula, which can hardly be treated, developed. And there was also the recommendation from them to apply for this soft mattress. And that’s what happened, I got it, and through the consultations and painkiller settings that were optimized in the day clinic, my everyday life has returned to normal”. (1001, 69y, diag.: cancer, para. 55)

Relief for relatives was also mentioned.

“But as I said, during her visits at the PDCC, I could also just do something for myself, I just- I also just went for a walk or I could do my work in peace, without constantly having the pressure then also, oh, I have to hurry, I have to go back to mommy and I have to do this. Or when the phone rang, you were startled, ‘Oh, what is it now?’. And that was simply not the case during her visits at the PDCC”. (1029B, 58y, diag. of pat.: cancer, para. 15)

#### Effects on relatives

Patients and relatives reported that their everyday life changed for the better since being connected to the PDCC and that this also had an impact on relatives.

“The children always know that I will come home again and that I will feel better. And my husband also knows that I’m in good hands and that I can discuss everything there if something´s wrong with me, and that they’re also very flexible about the medication and work together with my doctors in a great way. So in everyday life that also gives US a lot of quality, yes”. (1020, 37y, diag.: cancer, para. 27)

Conversations with staff and/or being treated by therapists themselves also had an effect on relatives.

“For me, it was first of all a psychological support, because I had just no idea what this means, this cancer and what we have to expect. (…) I understood what condition my husband was in. And even though I was there the whole time, I still felt it was a relief, because I saw that, yes, my husband was receiving good care, which we couldn’t have received at home”. (1031B, 52y, diag. of pat.: cancer, para. 37)

Relatives noticed relief from the PDCC in their everyday life.

“And it also relieves ME, because so these miserable debates about what to do now and how and why and whether she should really still take her medication, these issues are then taken away from me”. (1046B, 52y, diag. of pat.: cancer, para. 43)

## Discussion

Our study showed that PDCCs may close a gap between inpatient and home palliative care for those who do not yet need 24-hours hospital care. Participants mentioned that hospital stays can even be delayed or prevented by the use of PDCCs. The PDCC-AA supported not only patients but provided respite care for relatives and family members. Therefore it is a healthcare structure that may be beneficial for patient groups with palliative care needs at an earlier stage of the serious disease.

To date, the establishment and development of PDCCs in the UK [2, 3] and in Germany have been rather unsystematic. Therefore, a current study is exploring the status of and demand for PDCCs and day hospices in Germany [13]. This study represents a comprehensive qualitative evaluation of one of the first German PDCCs. From the perspective of those affected, the PDCC provides good palliative care and conveys confidence in dealing with one’s own illness. Hospital stays are delayed. An alternative form of care does not appear to be available to the interviewees in this form.

Due to missing guidelines, the PDCC-AA provides care to patients and family members to the best of their abilities. To support the development of further PDCCs, Terjung et al. describe a need for further research to determine necessary admission criteria and develop regional palliative care networks to foster and guide the establishment of PDCCs [14, 15]. Schuler (2019) criticizes deficits of early integration palliative care (EIPC) for oncological patients and missing evidence on the topic [16]. Available treatment alternatives, however, provide hints for EIPC at PDCC-AA. Our results show that attendance at the PDCC had a positive impact on both patients and relatives. This validates findings from Stevens et al. (2011), concluding that patients with palliative needs find attending a PDCC a valuable experience that allows them to find support in a restorative and safe environment [17].

Even though the data showed that most patients in the PDCC-AA had tumor diseases, the PDCC is of course “open” to non-tumor patients (e.g., patients with neurodegenerative diseases) and treatment will possibly have the same positive effect. However, our data match those found by Terjung et al. (2021), showing that a “typical” palliative day care patient is white, over 65 years old and has a cancer diagnosis. In their scoping review, they reported only a few studies treating patients with non-malignant diseases [14].

As Vries et al. (2012) observed, referring physicians have medical reasons for referring patients to PDCCs or day hospices. However, patients value the social component most of all [18]. The services offered by the PDCC under study include not only palliative medical and nursing treatment, examinations and interdisciplinary therapies but also psychosocial care. Particularly important and worth emphasizing is the connection to a hospital, so the structures there can be used (e.g., surgery in the case of wound care or the consultation of colleagues), which will possibly result in cost savings. In contrast, Douglas et al. (2005) found that the specific therapies and medical treatments were most important for patients when attending a PDCC. Social care was perceived as less essential. Whether the length of stay was a full day or a few hours was not as important to patients as receiving therapies and medical treatments [19]. Our results showed that patients and relatives valued the multidisciplinary teamwork approach, co-therapies (physical therapy, art therapy, and music therapy) and the social and psychological support. This is in line with research by Bradley et al. (2011) showing that patients emphasize the importance of person-centred care that reduces isolation, increases social support, encourages communication and provides activities [20]. As a terminal illness can cause multiple losses to quality of life, such as physical impairment, loss of social interaction, low mood and increased burden on relatives, visiting the PDCC brought changes to the everyday life of both patients and their families. Participants described a change in mood and a sense of confidence and security coping with their illness. In their cross-sectional survey, Dierickx et al. (2021) found reduced caregiver burden, a benefit of social support and contacts and enabling patients to live at home for as long as possible [21]. This is also shown by our results, where relatives valued the opportunity to have time for themselves and felt relief from the PDCC-AA in their everyday life.

### Strengths/limitations

Limitations of qualitative content analysis and thus from our study are e.g., that the research quality depends very much on the individual skills of the researcher. AM is trained and experienced in qualitative data collection and therefore this limitation is largely obsolete. AM and JS both coded the data and conducted consistency checks to ensure interpretations of data are consistent and transparent. Research bias was further reduced by engaging other researchers within a research workshop to discuss the categories.

Since data are usually collected from a few individuals findings cannot be generalized. However, the goal of most qualitative studies is not intended to generalize but rather to provide a contextualized understanding only relevant to a small group of population. We believe that the power of data based on patient and caregiver experience is sometimes more compelling and useful, especially in the field of health services research.

Although gatekeeping by healthcare professionals is a potential risk in palliative care research and occurs often by the general assumption of vulnerability of patients and the perceived need to protect patients [22], we were able to recruit a large sample size into our study. To further indicate the trustworthiness of our results, representative quotations are presented for each subcategory [23].

One possible limitation is that eligible patients were identified by their physicians, which may have biased our results towards positive narrations, although there were also isolated negative annotations. Another caveat relates to the overrepresentation of cancer patients. This might have biased our results, as oncological patients are often integrated sooner into palliative care and might therefore express their expectations and experiences in a different manner than other patient groups. Furthermore, member checking has not been done due to time and financial reasons and to avoid further burdening of the participants.

Unfortunately, it is not clear how many patients refused to participate. The study design did not allow for a structured comparison of those patients who were invited and those who participated/refused.

## Conclusion

The PDCC-AA may close a gap between inpatient and home palliative care. Findings may indicate that a PDCC supplements a palliative care network. It is appreciated very much by patients and relatives, as they mention that it prevents otherwise necessary hospital stays. The findings may hint at the need for further research, but they do not provide conclusive evidence that it reduces hospital admissions or length of stay. This evaluation will hopefully contribute to the establishment and development of further PDCCs in Germany by providing experiences and expertise as well as key figures for the demand, accessibility and practice of a PDCC and its benefits for patients and families.

## Data Availability

The datasets generated and/or analyzed during the current study are not publicly available due to limitations of ethical approval involving the participants' data and anonymity. They are available from the corresponding author on reasonable request.

## Declarations

Participants were provided with oral and written information about the study and provided written informed consent.
